# Recent Fast Food Consumption and Bisphenol A and Phthalates Exposures among the U.S. Population in NHANES, 2003–2010

**DOI:** 10.1289/ehp.1510803

**Published:** 2016-04-13

**Authors:** Ami R. Zota, Cassandra A. Phillips, Susanna D. Mitro

**Affiliations:** Department of Environmental and Occupational Health, Milken Institute School of Public Health, George Washington University, Washington, DC, USA

## Abstract

**Background:**

Phthalates and bisphenol A (BPA) are widely used industrial chemicals that may adversely impact human health. Human exposure is ubiquitous and can occur through diet, including consumption of processed or packaged food.

**Objective:**

To examine associations between recent fast food intake and BPA and urinary metabolites of di(2-ethylhexyl) phthalate (ΣDEHPm) and diisononyl phthalate (DiNPm) among the U.S. population.

**Methods:**

We combined data on 8,877 participants from the National Health and Nutrition Examination Survey (NHANES 2003–2010). Using 24-hr dietary recall data, we quantified: a) fast food intake [percent of total energy intake (TEI) from fast food]; b) fast food-derived fat intake (percent of TEI from fat in fast food); and c) fast food intake by food group (dairy, eggs, grains, meat, and other). We examined associations between dietary exposures and urinary chemical concentrations using multivariate linear regression.

**Results:**

We observed evidence of a positive, dose–response relationship between fast food intake and exposure to phthalates (p-trend < 0.0001) but not BPA; participants with high consumption (≥ 34.9% TEI from fast food) had 23.8% (95% CI: 11.9%, 36.9%) and 39.0% (95% CI: 21.9%, 58.5%) higher levels of ΣDEHPm and DiNPm, respectively, than nonconsumers. Fast food-derived fat intake was also positively associated with ΣDEHPm and DiNPm (p-trend < 0.0001). After adjusting for other food groups, ΣDEHPm was associated with grain and other intake, and DiNPm was associated with meat and grain intake.

**Conclusion:**

Fast food may be a source of exposure to DEHP and DiNP. These results, if confirmed, could inform individual and regulatory exposure reduction strategies.

**Citation:**

Zota AR, Phillips CA, Mitro SD. 2016. Recent fast food consumption and bisphenol A and phthalates exposures among the U.S. population in NHANES, 2003–2010. Environ Health Perspect 124:1521–1528; http://dx.doi.org/10.1289/ehp.1510803

## Introduction

Phthalates are a class of high production volume industrial chemicals that are ubiquitously used in commerce. High-molecular-weight phthalates, such as di(2-ethylhexyl) phthalate (DEHP), are used as plasticizers to impart flexibility in polyvinyl chloride (PVC) materials such as food packaging, flooring, and medical devices (U.S. EPA 2012). In recent years, other phthalates, including diisononyl phthalate (DiNP), have been replacing DEHP in these applications due, in part, to legislation limiting the use of DEHP in certain applications (European Chemicals Agency 2012). Bisphenol A (BPA) is a high production volume chemical used to make polycarbonate plastics and epoxy resins, found in food and beverage cans as well as thermal receipt paper (Calafat et al. 2008).

Phthalates and BPA can leach, migrate, or off-gas from products over time and enter the human body via ingestion, inhalation, and dermal absorption. Once in the body, phthalates and BPA are quickly metabolized and excreted in urine, with elimination half-lives less than 24 hr (Johns et al. 2015; Vandenberg et al. 2007). Human exposure to these chemicals is widespread (CDC 2013). Urinary metabolites of DEHP and DiNP are detected in 98% of the US general population (Zota et al. 2014) with higher exposures observed in children (Koch et al. 2004; Wittassek et al. 2011). Urinary metabolites of BPA are detected in 90% of the U.S. population with higher exposures observed in non-Hispanic Blacks, children, females, and those of lower socioeconomic status (Calafat et al. 2008; Nelson et al. 2012).

Experimental animal studies demonstrate that DEHP and DiNP have endocrine-disrupting properties because of their anti-androgenic effects on the male reproductive system (National Research Council 2008). Human exposure to DEHP has been associated with adverse reproductive, neurobehavioral, and respiratory outcomes in children (Braun et al. 2013; Ejaredar et al. 2015) and metabolic disease risk factors such as insulin resistance in adolescents and adults (James-Todd et al. 2012; Attina and Trasande 2015). Though epidemiologic evidence of DiNP is less complete, recent studies report associations between exposure and similar health outcomes including adverse respiratory and metabolic outcomes in children (Bertelsen et al. 2013; Attina and Trasande 2015). BPA is also a suspected endocrine disrupter, and experimental and human evidence suggest that BPA is a reproductive toxicant (Peretz et al. 2014). In addition, prenatal BPA exposure has also been associated with adverse neurobehavioral outcomes in children (Mustieles et al. 2015).

Given the concern over chemical toxicity, it is important to identify modifiable sources of exposure that may be targeted for exposure reduction strategies. Simulated exposure modeling, observational epidemiologic studies, and intervention studies all suggest that diet is an important exposure pathway for both high-molecular-weight phthalates and BPA (Carwile et al. 2011; Geens et al. 2012; Rudel et al. 2011; Serrano et al. 2014; Trasande et al. 2013; Wormuth et al. 2006). For example, a recent review of dietary and non-dietary exposures to BPA concluded that food sources contributed to more than 90% of overall BPA exposure among non-occupationally exposed individuals (Geens et al. 2012), and a second study that examined five individuals in Bochum, Germany fasting for 48 hr found that diet was the most significant source for DEHP and DiNP exposures as well (Koch et al. 2013). Food is likely contaminated with phthalates and BPA during processing (Cao 2010; Geens et al. 2012). Phthalates have been shown to leach into food from PVC in materials like tubing used in the milking process, lid gaskets, food preparation gloves, conveyor belts and food packaging materials (Cao 2010; Serrano et al. 2014). In fact, an intervention study reported that urinary BPA and DEHP were reduced by 66% and 53–56%, respectively, when participants’ diets were restricted to food with limited packaging (Rudel et al. 2011). Foods high in fat, such as dairy and meat, may be more contaminated by high-molecular-weight phthalates that are more lipophilic such as DEHP (Serrano et al. 2014).

Fast food may be an important source of exposure to phthalates and BPA because it is highly processed, packaged, and handled. A recent study of children 1–5 years old found that those who ate one or more fast food meals a week had greater DiNP and butylbenzyl phthalate exposures than did those who ate less than one meal a week (Watkins et al. 2014); however, that study was limited by the small age range of participants and the imprecise measure of fast food consumption. In this study, we investigated the association between recent fast food consumption (derived from 24-hr dietary recall data) and exposure to high-molecular-weight phthalates (DEHP and DiNP) and BPA in the U.S. population using data from the National Health and Nutrition Examination Survey (NHANES). We focus on DEHP and DiNP because previous work shows that estimated dietary exposures to these phthalates are higher than other commonly studied phthalates (Sakhi et al. 2014). We hypothesized that increased consumption of fast food will be associated with higher urinary levels of BPA and the metabolites of these two phthalates.

## Methods

### Study Population

We used data from the 2003–2004, 2005–2006, 2007–2008, and 2009–2010 cycles of NHANES, a nationally representative survey and physical examination of the civilian, non-institutionalized U.S. population conducted by the Centers for Disease Control and Prevention (CDC). The study sample included all participants ≥ 6 years old who completed a 24-hr dietary recall survey and provided a urine sample for phthalate or BPA analysis. The National Center for Health Statistics Research Ethics Review Board approved the study protocol. All participants gave informed consent; parents or guardians provided consent for participants < 18 years of age.

There were 10,506 participants with urinary measurements of phthalate metabolites and creatinine. We sequentially excluded participants who did not self-identify as non-Hispanic White, non-Hispanic Black, or Mexican American/Hispanic (*n* = 495); who were missing information on household income (*n* = 702); who were missing information on body mass index (BMI; *n* = 89); or who were missing kilocalorie data (*n* = 343) resulting in a final sample size of 8,877 study participants for the DEHP analyses. The final sample size for the DiNP analyses was 6,629 because the DiNP oxidative metabolite, monocarboxyoctyl phthalate (MCOP), was not measured in 2003–2004. Most chemical analytes are measured in approximately one-third of the overall NHANES population, and phthalate metabolites and BPA were measured in different subpopulations. There were 10,418 participants with urinary BPA measurements. Using our sequential exclusion criteria, we removed 1,626 participants and an additional 3 participants who were missing creatinine measures. The final sample size for BPA analyses was 8,789 study participants.

### Urinary Chemical Analysis

Phthalate metabolites and total BPA (free plus conjugated species) were measured in spot urine samples collected during the in-person exam at the Mobile Examination Center (MEC) and stored at –20°C until they were shipped to the CDC’s National Center for Environmental Health (Atlanta, GA) for analysis. Analytical methods have been described in detail elsewhere (CDC 2013). Briefly, chemical analytes were quantified in urine using solid-phase extraction coupled online with high-performance liquid chromatography and tandem mass spectrometry and expressed as wet weights (ng/mL). The limits of detection (LOD) ranged from 0.2 to 1.2 ng/mL for the phthalate metabolites and varied across study cycles. Therefore, we assumed the maximal LOD for each phthalate metabolite in our analysis to facilitate aggregation of data across study cycles (Zota et al. 2014). The LOD for BPA was 0.4 ng/mL for all study cycles. For both phthalates and BPA, concentrations below the LOD were substituted with the LOD divided by the square root of two. All analytes were detected in more than 90% of the study population except for mono(2-ethylhexyl) phthalate (MEHP; 64% > LOD).

To approximate DEHP exposure, we calculated a summary metric (ΣDEHPm) equal to the molar sum of four DEHP metabolites: MEHP, mono(2-ethyl-5-hydroxyhexyl) phthalate (MEHHP), mono(2-ethyl-5-oxohexyl) phthalate (MEOHP), and mono(2-ethyl-5-carboxypentyl) phthalate (MECPP) using an approach previously described in Zota et al. (2014). DiNP exposure was characterized by its oxidative metabolite MCOP (referred to as DiNPm throughout the manuscript).

### Dietary Assessment

The primary 24-hr dietary recall interview was administered in person at the MEC by dietary interviewers. Detailed protocols are described elsewhere and are briefly summarized here (CDC 2002). Survey participants ≥ 12 years old completed the dietary interview on their own, and proxy-assisted interviews were conducted with children 6–11 years old. During the interview, participants were prompted to report an uninterrupted listing of all foods and beverages consumed in a 24-hr period the day before the interview (midnight to midnight). This information was used to estimate the types and amounts of foods and beverages consumed as well as intakes of energy, nutrients, and other food components from those foods and beverages. Analysts at the National Center for Health Statistics (NCHS) in partnership with the U.S. Department of Agriculture’s (USDA) Food Surveys Research Group processed the intake data using the USDA’s Food and Nutrient Database for Dietary Studies (CDC 2002, 2007). Their output included publicly available datasets that summarize total nutrients for each participant as well as individual food profiles.

The dietary recall interview also asked about the source of each food item. Fast food was defined as food obtained from restaurants without waiter/waitress service, or from pizza restaurants regardless of waiter/waitress service. All carryout and delivery food was also considered fast food. Foods from all other sources, including restaurants with waiters/waitresses, bars, taverns, lounges, vending machines, and mail or packaged foods, were not considered fast food (CDC 2007). For this analysis, we extracted 24-hr intake of kilocalories and grams of fat, in total and from fast food specifically, by participant. We also extracted kilocalories of fast food by the following food groups as designated by the USDA: dairy, eggs, grains, meat, other (examples of fast food items in each food group are provided in Table 1).

**Table 1:** Common fast food items classified by their USDA-designated food groups.

Food group	Example foods in the group
Dairy	Milk, yogurt, milkshakes, smoothies, whipped cream, half-and-half, sour cream, ice cream, pudding, white sauce, all cheeses
Eggs	Whole boiled eggs, omelets, scrambled, breakfast sandwiches (e.g., egg and sausage on English muffin)
Grains	Bread, rolls, cake, croutons, biscuits, corn bread, hush puppies, tortillas, taco shells, muffins, cheesecake, cookies, pie, doughnuts, chips, pancakes, waffles, noodles, rice dishes, all burritos, all enchiladas, all tacos, all nachos, all quesadillas, all pizza, calzones, egg rolls, noodle soups
Meat	Hamburger, cheeseburger, chicken nuggets, chicken fillet sandwich, beef (steak, brisket, corned, ground, pastrami, jerky), ham, pork, bacon, lamb, chicken (breast, thigh, wings), hot dogs, sausage, bologna, pepperoni, fish, shrimp, chili with meat, turkey, gravy
Other	Beans (baked, refried), soy sauce, bacon bits, nuts, raw fruit and juice, guacamole, vinegar, potato (includes fries, chips, hash brown, mashed), vegetables, salads, sauces (ketchup, salsa, barbecue sauce, mustard, salad dressing, honey mustard, mayo, sweet and sour sauce), onion rings, pickles, olives, butter, margarine, syrups (chocolate, maple, honey), jelly, sugar, beverages (coffee, tea, soda, fruit-flavored drinks)

### Statistical Analysis

Analyses were conducted in SAS (version 9.3; SAS Institute Inc., Cary, NC). Because we combined four survey cycles, we calculated new sample weights for each participant according to the NCHS analytical guidelines (CDC 2002). The degrees of freedom for our study sample were calculated by subtracting the number of clusters in the first level of sampling (strata) from the number of clusters in the second level of sampling (PSUs) (National Center for Health Statistics 2006). Based on our degrees of freedom, we used the following critical values from the *t* distribution for the calculation of all confidence intervals (CIs): DEHPm (1.99); DiNPm (2.01); and BPA (1.99). All analyses were adjusted for the non-random sampling design and the sample population weights. A (two-sided) *p*-value < 0.05 was considered statistically significant.

Consumption of fast food was modeled several ways. First, we compared those who ate any fast food to those who did not. Second, we calculated fast food intake as the percent of total energy intake (TEI) from fast food in the 24-hr period. Third, we calculated fast food-derived fat intake as the percent of TEI from kilocalories of fat in fast food in the 24-hr period. Intake of fast food and fast food-derived fat were modeled as the following three categories: none, low, and high; where the low and high categories are divided at the weighted median of the exposed population. As a sensitivity analysis, we separately adjusted our final fast food intake models for intake from vending machines and from restaurants (percent of TEI; modeled as none, low, high) to account for other potential sources of packaged and processed food.

We additionally examined fast food intake by the five USDA food groups. First, we examined each food group separately, dividing the percent of TEI from fast food for each food group into three categories (none, low, high). Second, to account for potential confounding by non-fast food sources, we adjusted each food group by its non-fast food counterpart (e.g., percent of TEI from fast food meat was adjusted for percent of TEI from non-fast food meat). Percent of TEI from non-fast food sources were also modeled as three categories (none, low, high). Third, to account for potential confounding by other food groups, we included all five of the fast food groups in the same model.

We used multivariate linear regression models to examine the association between fast food consumption and urinary chemical concentrations. We performed natural log-transformation of chemical concentration data prior to regression analysis to improve normality and stabilize the variance. All regression models included natural log–transformed urinary creatinine [a marker of urine dilution (Barr et al. 2005)] and the following demographic variables: age (continuous); sex, race/ethnicity [non-Hispanic white, non-Hispanic black, or Hispanic (including Mexican American)], BMI [underweight (< 18.5), normal (18.5–24.99), overweight (25–29.99) and obese (> 30)], poverty:income ratio (PIR; the ratio of household income to poverty threshold adjusted to family size and inflation; [< 1 (i.e., beneath the poverty threshold), 1–3, and > 3], and NHANES survey cycle (2003–2004, 2005–2006, 2007–2008, 2009–2010).

From these regression models, we estimated: *a*) percent difference in urinary chemical concentrations by fast food consumption as (*e* ^(β)^ – 1) × 100% with 95% CIs estimated as (*e* ^(β ± critical value × SE)^ – 1), where β and SE are the estimated regression coefficient and standard error, respectively; and *b*) least squares geometric means of urinary chemical concentrations by fast food consumption as *e* ^(least squares means)^ with 95% CIs as *e* ^(least squares mean ± critical value × SE)^, where the least square means is the mean of urinary chemical concentrations by fast food intake after adjustment for covariates. When exposure was modeled as three categories, percent differences were estimated by comparing each of the upper two groups to the lowest group. Additionally, a test for trend was performed by modeling the integer value of each exposure category (i.e., 0, 1, 2) as an ordinal term, and using its *p*-value as a test of departure from the null hypothesis of no linear trend. We tested for potential effect modification by age, sex, race/ethnicity, and household income by including multiplicative interaction terms in the statistical models. To further assess whether our results were specific to fast food, we also examined associations of TEI (tertile categories: low, medium, high) and total fat intake (percent of TEI; tertile categories: low, medium, high) with urinary chemical concentrations.

## Results

Approximately one-third of the 8,877 participants reported consuming fast food on the day prior to their urine sample collection. Participants who ate fast food were more likely to be < 40 years old, male, and non-Hispanic black and to have higher TEI and total fat intake. Fast food consumers had higher levels of ΣDEHPm, DiNPm, and BPA than did nonconsumers. This difference was statistically significant for phthalates but not for BPA (Table 2).

**Table 2:** Demographic characteristics and urinary chemical concentrations by recent fast food consumption in the U.S. general population, NHANES 2003–2010 (*n *= 8,877).

Variable	Fast food consumption		
	Yes (*n *= 3,095)	No (*n *= 5,782)	*p-*Value
Characteristic, % (SE)^*a,b *^
Age (years)			< 0.0001
6–11	35.1 (2.3)	64.9 (2.3)
12–19	42.6 (1.4)	57.4 (1.4)
≥ 20	32.8 (0.8)	67.2 (0.8)
Sex			< 0.0001
Male	37.2 (1.1)	62.8 (1.1)
Female	31.4 (0.9)	68.6 (0.9)
Race/ethnicity			< 0.0001
Hispanic (incl. Mexican-American)	35.0 (1.0)	65.0 (1.0)
Non-Hispanic white	32.5 (1.0)	67.5 (1.0)
Non-Hispanic black	43.8 (1.2)	56.2 (1.2)
Body mass index (BMI) (kg/m^2^)			0.526
< 18.5	35.8 (2.1)	64.2 (2.1)
18.5–25	33.2 (1.1)	66.8 (1.1)
25–30	34.1 (1.4)	65.9 (1.4)
≥ 30	35.1 (1.2)	64.9 (1.2)
Poverty:income ratio (PIR)			0.992
< 1	34.1 (1.2)	65.9 (1.2)
1–2.99	34.3 (1.3)	65.7 (1.3)
≥ 3	34.2 (1.1)	65.8 (1.1)
NHANES survey period			0.041
2003–2004	35.6 (1.5)	64.4 (1.5)
2005–2006	36.1 (1.3)	63.9 (1.3)
2007–2008	34.6 (2.1)	65.4 (2.1)
2009–2010	30.7 (0.9)	69.3 (0.9)
Energy and nutrient, GM (GSE)^*c*^
Total energy intake (TEI; kilocalories)	2,225 (24.0)	1,878 (16.0)	< 0.0001
Total fat intake (% of TEI)	33.7 (0.2)	31.2 (0.4)	< 0.0001
Chemical concentrations (ng/mL), GM (GSE)^*c*^
∑DEHPm	83.6 (3.5)	59.1 (2.0)	< 0.0001
DiNPm	10.1 (0.7)	7.0 (0.3)	< 0.0001
BPA	2.4 (0.1)	2.0 (0.05)	0.142
∑DEHPm, molar sum of four DEHP metabolites; DiNPm, DiNP metabolite (MCOP); GM, geometric mean; GSE, geometric standard error; SE, standard error. Note: Sample size for DINPm is 6,629 (data unavailable for 2003–2004), and for BPA, it is 8,789. ^***a***^Percentages are weighted. ^***b***^Differences in demographic characteristics by fast food consumption were evaluated using chi-square test of independence. ^***c***^Differences in energy and nutrients and urinary chemical concentrations by fast food consumption were evaluated using linear regression adjusting for urinary creatinine.			

We observed evidence of a positive, dose–response association between fast food intake and ΣDEHPm (*p* for trend < 0.0001) (Table 3). Compared to nonconsumers, low consumers had 15.5% (95% CI: 6.3%, 25.6%) and high consumers had 23.8% (95% CI: 11.9%, 36.9%) higher levels of ΣDEHPm, respectively. Fast food-derived fat intake was also significantly associated with ΣDEHPm (*p* for trend < 0.0001) but smaller in magnitude than the association with fast food intake. For example, high consumers of fast food-derived fat had 18.9% (95% CI: 8.9%, 29.9%) higher levels of ΣDEHPm compared to nonconsumers. Furthermore, total fat intake (of all foods) but not TEI was associated with ΣDEHPm. Consumers in the highest tertile had 17.3% (95% CI: 9.2%, 26.0%) higher levels of ΣDEHPm, respectively, than consumers in the lowest tertile (Table 3).

**Table 3:** Association between recent fast food consumption and urinary chemical concentrations in the U.S. general population, NHANES 2003–2010.

Dietary intake	∑DEHPm (*n *= 8,877)		DiNPm (*n *= 6,629)		BPA (*n *= 8,789)	
	*n*	Percent difference (95% CI)	*n*	Percent difference (95% CI)	*n*	Percent difference (95% CI)
TEI (kcal)^*a*^
Low (54–1,671)	3,158	Referent	2,417	Referent	3,114	Referent
Moderate (1,672–2,413)	2,953	4.3 (–3.7, 13.1)	2,245	6.2 (–2.5, 15.6)	2,983	–1.5 (–7.6, 4.9)
High (2,414–13,133)	2,766	3.6 (–4.5, 12.3)	1,967	6.5 (–2.0, 15.8)	2,692	–3.9 (–10.0, 2.5)
*p* for trend		0.39		0.13		0.23
Fast food intake (% of TEI)^*b*^
None (0)	5,782	Referent	4,354	Referent	5,750	Referent
Low (0.08–34.8)	1,500	15.5 (6.3, 25.6)**	1,114	24.8 (12.9, 37.9)**	1,461	1.1 (–4.8, 7.4)
High (34.9–100)	1,595	23.8 (11.9, 36.9)**	1,161	39.0 (21.9, 58.5)**	1,578	3.6 (–2.8, 10.5)
*p* for trend		< 0.0001		< 0.0001		0.28
Total fat intake (% of TEI)^*a*^
Low (0.0–29.9)	3,037	Referent	2,262	Referent	2,976	Referent
Moderate (30.0–37.0)	2,957	8.6 (1.4, 16.3)*	2,228	11.2 (0.2, 23.5)*	3,000	–0.8 (–6.4, 5.2)
High (37.1–74.7)	2,883	17.3 (9.2, 26.0)**	2,139	9.9 (–0.3, 21.1)	2,813	0.6 (–6.0, 7.5)
*p* for trend		< 0.0001		0.06		0.87
Fast food-derived fat intake (% of TEI)^*b*^
None (0)	5,803	Referent	4,370	Referent	5,769	Referent
Low (0.003–14.1)	1,489	18.9 (8.9, 29.9)**	1,094	27.1 (14.4, 41.3)**	1,461	0.5 (–5.9, 7.3)
High (14.2–59.8)	1,585	19.3 (9.0, 30.7)**	1,165	35.0 (18.9, 53.3)**	1,559	4.1 (–2.6, 11.4)
*p* for trend		< 0.0001		< 0.0001		0.25
∑DEHPm, molar sum of four DEHP metabolites; DiNPm, DiNP metabolite (MCOP); TEI, total energy intake. Note: Adjusted for age, sex, race/ethnicity, BMI, PIR, NHANES survey cycle, and urinary creatinine. ^***a***^Tertiles categories (low, moderate, high) were calculated using the weighted distribution of the entire study population (*n *= 8,877). ^***b***^Low and high categories are divided at the weighted median of the exposed population (*n *= 8,877). *p < 0.05. **p < 0.01.						

Similar to ΣDEHPm, there was evidence of a positive dose–response association between fast food intake and DiNPm (*p* for trend < 0.0001) (Table 3). Compared to nonconsumers, low consumers had 24.8% (95% CI: 12.9%, 37.9%) and high consumers had 39.0% (95% CI: 21.9%, 58.5%) higher levels of DiNP, respectively. The association with fast food-derived fat intake and DiNPm was smaller in magnitude than the association with fast food intake. Furthermore, total fat intake but not TEI was associated with DiNPm; however, this association was not monotonic and only the middle tertile estimate was significant (Table 3).

In sensitivity analyses, adjustment for restaurant intake increased the associations between fast food intake and ΣDEHPm and DiNP by 10% to 20% (see Table S1) while there was virtually no change in the main effects after adjustment for vending machine intake.

Next we calculated the association between fast food intake specific to each USDA food group with ΣDEHPm. All food groups were frequently consumed except for eggs, which were consumed in fast food meals by only 2% of the population. When each food group was modeled individually, we observed significant associations for all food groups except for eggs, although none of the associations were monotonic (Table 4; Model 1). These associations were virtually unchanged when each food group model was further adjusted for intake of non-fast food sources from the same food group (Table 4; Model 2). However, when all the food groups were examined together in the same model, only the individual categories of high grain and low other intake remained significant. Furthermore, the association between grain intake and ΣDEHPm became monotonic with a significant test for trend (*p* for trend = 0.01) (Table 4, Model 3).

**Table 4:** Association between recent fast food consumption by food group and urinary concentrations of ∑DEHPm in the U.S. general population, NHANES 2003–2010 (*n *= 8,877).

Fast food intake (% of TEI) by food group^*a*^	Model 1^*b*^		Model 2^*c*^		Model 3^*d*^	
	Percent difference (95% CI)	*p* for trend	Percent difference (95% CI)	*p* for trend	Percent difference (95%CI)	*p* for trend
Dairy
None (0%) (*n *= 8,138)	Referent		Referent		Referent
Low (≤ 5.6) (*n *= 351)	21.2 (–1.3, 48.7)		21.4 (–1.2, 49.2)		2.3 (–18.5, 28.3)
High (> 5.6) (*n *= 388)	23.0 (0.2, 51.0)*	0.01	23.6 (0.9, 51.3)*	0.01	8.3 (–12.8, 34.6)	0.32
Eggs
None (0%) (*n *= 8,725)	Referent		Referent		Referent
Low (≤ 12.2) (*n *= 79)	28.3 (–6.6, 76.1)		30.2 (–5.0, 78.2)		11.2 (–19.0, 52.5)
High (> 12.2) (*n *= 73)	8.4 (–16.4, 40.5)	0.16	10.2 (–15.2, 43.1)	0.12	0.7 (–23.7, 32.9)	0.58
Grains
None (0%) (*n *= 7,139)	Referent		Referent		Referent
Low (≤ 18.1) (*n *= 844)	21.3 (7.1, 37.4)**		21.9 (8.0, 37.6)**		5.5 (–8.4, 21.4)
High (> 18.1) (*n *= 894)	20.3 (8.4, 33.3)**	< 0.0001	21.5 (9.3, 35.0)**	< 0.0001	11.9 (0.7, 24.4)*	0.01
Meat
None (0%) (*n *= 6,790)	Referent		Referent		Referent
Low (≤ 17.8) (*n *= 1,020)	22.9 (11.7, 35.4)**		24.5 (12.9, 37.3)**		6.9 (–5.0, 20.4)
High (> 17.8) (*n *= 1,067)	16.4 (7.3, 26.4)**	< 0.0001	18.3 (8.3, 29.2)**	< 0.0001	5.4 (–4.9, 16.7)	0.13
Other
Non (0%) (*n *= 6,670)	Referent		Referent		Referent
Low (≤ 10.4) (*n *= 1,071)	25.5 (14.9, 37.0)**		25.4 (14.7, 37.2)**		14.1 (2.7, 26.9)*
High (> 10.4) (*n *= 1,136)	20.7 (9.8, 32.6)**	< 0.0001	20.6 (9.1, 30.4)**	< 0.0001	10.1 (–2.2, 24.0)	0.05
∑DEHPm, molar sum of four DEHP metabolites; TEI, total energy intake. ^***a***^Low and high categories are divided at the weighted median of the exposed group. ^***b***^Adjusted for age, sex, race/ethnicity, BMI, PIR, NHANES survey cycle, and urinary creatinine. ^***c***^Model 1 with additional adjustment of intake (% of TEI) from non-fast food group counterpart (e.g., fast food dairy intake adjusted for non-fast food dairy intake, fast food egg intake adjusted for non-fast food egg intake, etc.). ^***d***^Model 1 with additional adjustment for fast food intake (% of TEI) of all other food groups. *p < 0.05. **p < 0.01.						

Similar to ΣDEHPm, we observed significant associations between DiNPm and all food groups except for eggs when each group was modeled individually (Table 5, Model 1). When each food group was further adjusted for intake of non-fast food sources from the same food group, associations were virtually unchanged (Table 5, Model 2). When all the food groups were examined together in the same model, we observed significant, monotonic associations for grain and meat intake (*p* for trend < 0.05). Those with high fast food grain and meat consumption had 21.9% (95% CI: 9.4%, 35.8%) and 20.1% (95% CI: 3.0%, 40.1%) higher levels of DiNPm, respectively, than did nonconsumers of those fast food groups (Table 5, Model 3).

**Table 5:** Association between recent fast food consumption by food group and urinary concentrations of DiNPm in the U.S. general population, NHANES 2005–2010 (*n *= 6,629).

Fast food intake (% of TEI) by food group^*a*^	Model 1^*b*^		Model 2^*c*^		Model 3^*d*^	
	Percent difference (95% CI)	*p* for trend	Percent difference (95% CI)	*p* for trend	Percent difference (95% CI)	*p* for trend
Dairy
None (0%) (*n *= 6,059)	Referent		Referent		Referent
Low (≤ 5.5) (*n *= 266)	19.3 (–2.6, 46.1)		19.3 (–2.0, 45.3)		–4.0 (–20.4, 15.9)
High (> 5.5) (*n *= 304)	23.2 (0.6, 50.8)*	0.02	22.3 (0.1, 49.5)*	0.02	4.0 (–14.7, 26.7)	0.79
Eggs
None (0%) (*n *= 6,518)	Referent		Referent		Referent
Low (≤ 11.3) (*n *= 54)	2.5 (–30.2, 50.5)		3.0 (–29.9, 51.2)		–13.7 (–42.7, 30.0)
High (> 11.3) (*n *= 57)	28.4 (–22.9, 113.9)	0.30	29.1 (–22.6, 115.5)	0.29	22.0 (–29.4, 110.8)	0.69
Grains
None (0%) (*n *= 5,377)	Referent		Referent		Referent
Low (≤ 17.8) (*n *= 612)	27.0 (9.3, 47.5)**		27.6 (9.5, 48.7)**		9.8 (–6.0, 28.2)
High (> 17.8) (*n *= 640)	30.5 (15.9, 47.0)**	< 0.0001	32.8 (17.4, 50.1)**	< 0.0001	21.9 (9.4, 35.8)**	0.001
Meat
None (0%) (*n *= 5,079)	Referent		Referent		Referent
Low (≤ 18.0) (*n *= 752)	31.5 (14.6, 50.8)**		29.8 (13.5, 48.5)**		17.0 (2.5, 33.6)*
High (> 18.0) (*n *= 798)	30.4 (13.0, 50.4)**	< 0.0001	28.1 (11.0, 47.7)**	0.0001	20.1 (3.0, 40.1)*	0.02
Other
None (0%) (*n *= 5,014)	Referent		Referent		Referent
Low (≤ 10.2) (*n *= 787)	26.1 (8.0, 47.3)**		25.2 (7.0, 46.5)**		6.1 (–8.6, 23.2)
High (> 10.2) (*n *= 828)	33.1 (15.3, 53.6)**	< 0.0001	31.2 (13.1, 52.3)**	0.0002	10.9 (–5.1, 29.6)	0.15
Abbreviations: DiNPm, Di-iso-nonyl phthalate metabolite; TEI, total energy intake. Note: DiNPm was not measured in NHANES 2003–2004. ^***a***^Low and high categories are divided at the weighted median among the exposed group within the DiNP subpopulation (*n *= 6,629). ^***b***^Adjusted for age, sex, race/ethnicity, BMI, PIR, NHANES survey cycle, and urinary creatinine. ^***c***^Model 1 with additional adjustment of intake (% of TEI) from non-fast food group counterpart (e.g., fast food dairy intake adjusted for non-fast food dairy intake, fast food egg intake adjusted for non-fast food egg intake). ^***d***^Model 1 with additional adjustment for fast food intake (% of TEI) of all other food groups. *p < 0.05. **p < 0.01.						

There was a monotonic, dose–response association between fast food meat and BPA in all three models (*p* for trend ≤ 0.01) (see Table S2). When all the food groups were examined together in the same model (see Table S2, Model 3), those with high fast food meat intake had 11.9% (95% CI: 2.0%, 22.7%) higher levels of BPA, respectively, than did nonconsumers of fast food meat. Low egg intake was significantly associated with BPA in all three models although the estimates for high egg intake were near the null and the linear test for trend was not significant. Lastly, the highest tertile for grain intake was negatively associated with BPA across all three models.

**Figure 1:**
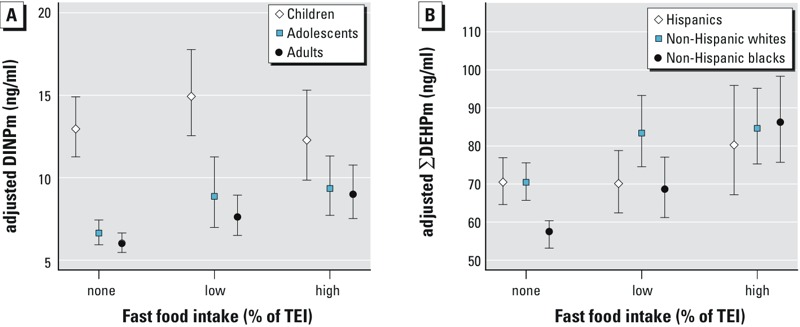
Association [LSGM (95% CI)] between fast food intake (percent of TEI) and urinary phthalate metabolite concentrations in the U.S. general population by (*A*) age for DiNPm (*n *= 6,629; *p_interaction_ *= 0.02) and (*B*) race/ethnicity for ΣDEHPm (*n *= 8,877; *p_interaction_ *= 0.04). Estimates in Figure A are from linear regression models of interactions between fast food intake and age group adjusted for urinary creatinine, sex, race/ethnicity, BMI, PIR, and NHANES survey cycle. Estimates in Figure B are from linear regression models of interactions between fast food intake and race/ethnicity adjusted for urinary creatinine, age, sex, BMI, PIR, and NHANES survey cycle. Corresponding percent change estimates are provided in Tables S3 and S4.

Tests for interaction by sex and household income were not significant. Tests for interaction by age were not significant for ΣDEHPm or BPA, but were significant for DiNPm (*p*_interaction_ < 0.05) (Figure 1; see also Table S3). Exposure to DiNPm was not associated with fast food intake in children, but was positively associated with fast food intake in adolescents and adults. Tests for interaction by race/ethnicity were not significant for DiNPm or BPA, but were significant for ΣDEHPm (*p*_interaction_ < 0.05) (Figure 1; see also Table S4). Exposure to ΣDEHPm was positively associated with fast food intake in all three racial/ethnic groups; however, the association did not reach statistical significance among Hispanics. Moreover, the highest tertile estimate for fast food intake was greater in magnitude for non-Hispanic blacks compared to non-Hispanic whites or Hispanics.

## Discussion

In this cross-sectional study of the U.S. population, we found a consistent, positive association between recent fast food consumption and phthalates exposure. Furthermore, there was evidence of a monotonic, positive dose–response; participants with high fast food intake had 20–40% higher urinary concentrations of phthalate metabolites than did nonconsumers. To our knowledge, this is the largest study to date on fast food consumption and biomarkers of environmental chemical exposure and the first to use a population-based sample.

We did not find an association between total fast food consumption and BPA. Our null finding may, in part, be explained by the fact that the intake of BPA from non-canned foods is likely minimal compared to BPA from canned foods and nondietary sources (e.g., contact with thermal receipts) (Geens et al. 2012). We did find a significant, monotonic association between fast food meat intake and BPA, which corresponds to the small but growing evidence suggesting that hamburgers may be a source of BPA exposure. In a 2008 total diet study in Quebec, Canada, levels of BPA in fast food composite samples were generally low (1–3 ng/g) except for in the hamburger (10.9 ng/g) (Cao et al. 2011). In a study of 491 Mexican-American pregnant women, those who consumed hamburgers three or more times a week had 20% higher urinary BPA levels than did nonconsumers of hamburgers (Quirós-Alcalá et al. 2013).

While fast food consumption was associated with exposure to both phthalates, our results highlight some important differences between DEHP and DiNP. The magnitude of the associations was 40–50% higher for DiNP than for DEHP. Moreover, associations for fast food-derived fat and DiNP were more pronounced than associations for TEI or total fat intake and DiNP suggesting DiNP contamination sources may be specific to fast food. In contrast, the magnitude of the association for highest intake of fast food-derived fat and total fat were similar for DEHP suggesting that some DEHP contamination sources may be common to all high fat foods. While many of the studies on dietary intake of phthalates do not include DiNP (Cirillo et al. 2011; Colacino et al. 2010; Schecter et al. 2013; Trasande et al. 2013), our results are consistent with a recent study of 296 children from Ohio ages 1–5 years that reported positive associations between fast food consumption and urinary metabolites of DiNP but not DEHP (Watkins et al. 2014). Additionally, a recent study of 10 phthalates in Norwegian food items found that DiNP was the most detected phthalate, and that estimated dietary exposures were also highest for DiNP (Sakhi et al. 2014). Our results show an interesting parallel with the changing trends in U.S. phthalate exposure as measured in biomonitoring data; between 2001 and 2010, ΣDEHP metabolites decreased by 37% and MCOP (DiNP metabolite) increased by 149% (Zota et al. 2014). Collectively, this work suggests that DiNP may be replacing DEHP in food contact materials. Future studies should further examine the human health effects associated with DiNP exposure and its potential synergy with other phthalates such as DEHP.

Our analysis of fast food consumption by food groups found that intake of grain items was significantly associated with DiNP and DEHP with evidence of a monotonic, positive dose response. Meat intake was also associated with DiNP and DEHP, but the association with DEHP did not remain significant after adjusting for other food groups. Many food monitoring studies report higher phthalate residues in high fat foods, including oils, meat, and dairy (Cao 2010; Serrano et al. 2014). In line with these data, some epidemiological studies show positive associations between consumption of meats, fats, and dairy products and DEHP (Serrano et al. 2014). The robust association between fast food grain items and both phthalates in our study may, in part, be a result of the way the food items were classified. The grains category was heterogeneous and included a wide variety of items (Table 1) such as bread, cake, pizza, burritos, rice dishes, and noodles. Similar to our results, however, the recent Norwegian food monitoring study also identified grains to be the largest contributor to estimated daily intake of DEHP and DiNP, suggesting that grains consumption may be a true source of exposures (Sakhi et al. 2014). For example, since grain products are found on the exterior of foods such as pizza or burritos, they may be in greater contact with packaging materials. Future research should further investigate phthalate content in specific fast food menu items or by fast food chains to further characterize dietary exposures to phthalates.

Our findings also suggest that associations between fast food intake and phthalate exposure are not uniform across the population. The association between DiNP and fast food was observed in adolescents and adults, but not in children 6–11 years old, potentially reflecting differences in exposure sources or behavior between groups. Furthermore, the association between fast food intake and DEHP exposure was more pronounced in blacks than in Hispanics, which may be linked to the higher consumption of fast food calories among blacks (see Table S4), or to differences in the types of fast food meals consumed between racial/ethnic groups. Future research could expand on these findings by examining how the contribution of dietary and non-dietary sources to phthalates exposures varies by race/ethnicity or socioeconomic status.

The complexity and variability of fast food production makes it difficult to identify the sources of high-molecular-weight phthalates, though some likely sources have been suggested, including PVC gloves, PVC tubing, and plastic packaging. Food monitoring and duplicate diet studies conducted in Japan found that use of disposable PVC gloves during the preparation and packaging of meals was a major source of dietary intake of DEHP and that sterilizing the gloves with alcohol increased DEHP migration (Tsumura et al. 2001a, 2001b). The same study team also demonstrated a decline in DEHP levels in prepared meals after the ban of DEHP in PVC gloves in Japan (Tsumura et al. 2003). PVC tubing and plastic packaging may play a role since many of the food items available at fast food restaurants are prepared in bulk quantities at central supply facilities and then shipped to individual restaurants where they are cooked, reheated, or assembled (Schlosser 2012). For example, an Italian study that compared levels of DEHP and di-*n*-butyl phthalate in school meals before and after the food was packaged found that packaging increased phthalate concentrations by more than 100% (Cirillo et al. 2011). Future research should further characterize the role of food production, processing, and handling in dietary phthalate exposure.

One main study limitation is the cross-sectional design of NHANES; thus, we cannot infer a causal relationship between fast food consumption and urinary phthalate metabolites. Future research should confirm our findings using a longitudinal study design that includes assessment of phthalates exposure in individuals before and after the consumption of fast foods. Other study limitations included the reliance on a single spot urine sample. While multiple urine samples or a 24-hr total urine sample is ideal, the collection of a single spot urine within 24 hr of the reported dietary consumption corresponds well with the short elimination half-lives of phthalates and BPA. We also relied on self-reported dietary data, which may have been prone to errors in reporting. However, the measurement error arising from both the urine sample collection and dietary recall is likely nondifferential. Lastly, the fast food category encompassed a wide variety of food items and establishments, which makes it difficult to isolate specific types of food production or menu items that may be underlying the observed associations. However, there are also advantages to use of NHANES dietary data. The dietary interview used a specific definition of fast food that eliminates subjectivity by the participant, and the broad array of food choices included in the fast food category increases the generalizability of our results.

There are several notable strengths to our study. To our knowledge, this is the first study to use NHANES dietary recall data to examine the association between fast food consumption and urinary measures of environmental chemicals in the U.S. population. This study design allowed us to evaluate the associations among a large, representative sample of the U.S. population that included children. Moreover, we were able to quantify population-level consumption of fast food using information on energy and nutrients. Finally, our findings were robust to sensitivity analyses including adjustment for other potential sources of processed or packaged food as well as several different categorizations of fast food intake.

## Conclusions

In conclusion, our findings suggest that fast food consumption may be a source of exposure to DEHP and DiNP, but not BPA, among the general population. These results, if confirmed in future longitudinal studies, may have great public health significance given the recommendations by various scientific and governmental bodies to limit exposure to phthalates due to concern over potential adverse health effects (Chronic Hazard Advisory Panel on Phthalates and Phthalate Alternatives 2014; U.S. EPA 2012). Despite public health interest in reducing exposures, few modifiable sources have been identified. Therefore, our results may represent an important step forward in individual and regulatory phthalate exposure reduction strategies while population health effects remain under study.

## Supplemental Material

(178 KB) PDF
