# Supplementary material for: Recent Fast Food Consumption and Bisphenol A and Phthalates Exposures among the U.S. Population in NHANES, 2003–2010
Source: Environ Health Perspect. 2016 Apr 13;124(10):1521–8. doi: 10.1289/ehp.1510803 (PMC5047792; doi:10.1289/ehp.1510803)
Supplement: (178 KB) PDF [file ehp.1510803.s001.acco.pdf]

Environ Health Perspect
DOI: 10.1289/ehp.1510803

Note to readers with disabilities: *EHP* strives to ensure that all journal content is accessible to all readers. However, some figures and Supplemental Material published in *EHP* articles may not conform to 508 standards due to the complexity of the information being presented. If you need assistance accessing journal content, please contact ehp508@niehs.nih.gov. Our staff will work with you to assess and meet your accessibility needs within 3 working days.

Supplemental Material

Recent Fast Food Consumption and Bisphenol A and Phthalates Exposures among the U.S. Population in NHANES, 2003–2010

Ami R. Zota, Cassandra A. Phillips, and Susanna D. Mitro

Table of Contents

Table S1. Association between recent fast food consumption and urinary chemical concentrations additionally adjusted for restaurant food and vending machine consumption in the US general population, NHANES 2003-2004

Table S2. Association between recent fast food consumption by food group and urinary concentrations of BPA in the US general population, NHANES 2003-2010 (n = 8,789)

Table S3. Association between recent fast food consumption and urinary concentrations of DiNPm stratified by age group in the US general population, NHANES 2005-2010 (n = 6,629)

Table S4. Association between recent fast food consumption and urinary concentrations of ∑DEHPm stratified by race/ethnicity in the US general population, NHANES 2003-2010 (n = 8,877)

Table S1. Association between recent fast food consumption and urinary chemical concentrations additionally adjusted for restaurant food and vending machine consumption in the US general population, NHANES 2003-2004[a,b]

	∑DEHPm (n = 8,877)		DiNPm (n = 6,629)		BPA (n = 8,789)	
Fast food consumption	*n*	Percent difference (95% CI)	*n*	Percent difference (95% CI)	*n*	Percent difference (95% CI)
Model 1: Fast food intake (% of TEI)						
None (0)	5782	Referent	4354	Referent	5750	Referent
Low (0.08 - 34.8)	1500	15.5 (6.3, 25.6)**	1114	24.8 (12.9, 37.9)**	1461	1.1 (-4.8, 7.4)
High (34.9 – 100)	1595	23.8 (11.9, 36.9)**	1161	39.0 (21.9, 58.5)**	1578	3.6 (-2.8, 10.5)
p for trend		<0.0001		<0.0001		0.28
Model 2: Model 1 + restaurant food intake (% of TEI)[c]						
None (0)	5782	Referent	4354	Referent	5750	Referent
Low (0.08 - 34.8)	1500	16.3 (7.3, 26.0)**	1114	24.6 (12.8, 37.8)**	1461	1.2 (-4.8, 7.5)
High (34.9 – 100)	1595	29.0 (16.3, 43.0)**	1161	42.7 (24.6, 63.4)**	1578	3.7 (-2.8, 10.6)
p for trend		<0.0001		<0.0001		0.27
Model 3: Model 1 + vending machine intake (% of TEI)[d]						
None (0)	5782	Referent	4354	Referent	5750	Referent
Low (0.08 - 34.8)	1500	15.4 (6.2, 25.5)**	1114	24.7 (12.7, 38.0)**	1461	1.1 (-4.8, 7.4)
High (34.9 – 100)	1595	23.6 (11.8, 36.7)**	1161	39.2 (22.1, 58.7)**	1578	3.6 (-2.9, 10.6)
p for trend		<0.0001		<0.0001		0.29

*p<0.05; **p<0.01

Abbreviations: ∑DEHPm = molar sum of four DEHP metabolites; DiNPm = DiNP metabolite (MCOP); TEI = total energy intake

[a] Fast food establishment defined as a restaurant with no waiter/waitress service; all pizza restaurants; and all carryout and delivery food. Restaurant defined as dining establishment with waiters/waitresses.

[b] All models adjusted for age, sex, race/ethnicity, BMI, PIR, NHANES survey cycle, and urinary creatinine.

[c] 1632 participants reported consuming any restaurant food during the study period.

[d] 328 participants reported consuming any vending machine food during the study period.

Table S2. Association between recent fast food consumption by food group and urinary concentrations of BPA in the US general population, NHANES 2003-2010 (n = 8,789)

	Model 1[b]		Model 2[c]		Model 3[d]	
Fast food intake (% of TEI) by food group[a]	Percent difference (95%CI)	*p* for trend	Percent difference (95%CI)	*p* for trend	Percent difference (95%CI)	*p* for trend
Dairy						
None (0%) (n = 8,032)	Referent		Referent		Referent	
Low (≤ 5.6) (n = 361)	-0.8 (-10.5, 9.9)		-1.5 (-11.4, 9.6)		-1.7 (-13.2, 11.2)	
High (> 5.6) (n = 396)	7.7 (-3.8, 20.5)	0.27	7.0 (-4.4, 19.6)	0.33	6.6 (-5.1, 19.7)	0.26
Eggs						
0% (n = 8,640)	Referent		Referent		Referent	
Low (≤ 11.3) (n = 71)	33.5 (4.8,70.1)*		33.9 (5.3, 70.5)*		34.6 (5.6, 71.4)*	
High (> 11.3) (n = 78)	6.5 (-23.0, 47.4)	0.25	6.8 (-22.7, 47.6)	0.24	9.5 (-21.1, 52.0)	0.20
Grains						
None (0%) (n = 7,095)	Referent		Referent		Referent	
Low (≤ 18.0) (n = 831)	2.0 (-5.5, 10.2)		0.9 (-6.6, 8.9)		-2.6 (-11.3, 6.9)	
High (> 18.0) (n = 863)	-8.2 (-15.2, -0.5)*	0.11	-10.1 (-17.4, -2.2)*	0.04	-9.3 (-16.2, -1.7)*	0.03
Meat						
None (0%) (n = 6,744)	Referent		Referent		Referent	
Low (≤ 18.1) (n = 1,010)	2.4 (-4.6, 9.9)		3.7 (-3.6, 11.6)		4.4 (-4.1, 13.6)	
High (> 18.1) (n = 1,035)	10.1 (2.2, 18.5)*	0.01	12.6 (4.2, 21.7)**	0.002	11.9 (2.0, 22.7)*	0.008
Other						
None (0%) (n = 6,631)	Referent		Referent		Referent	
Low (≤ 10.5) (n = 1,051)	2.8 (-4.8, 10.9)		3.0 (-4.3, 10.9)		0.0 (-8.7, 9.49)	
High (> 10.5) (n = 1,107)	1.5 (-4.6, 8.0)	0.50	2.1 (-3.9, 8.4)	0.38	-3.8 (-11.6, 4.8)	0.32

*p<0.05; **p<0.01

Abbreviations: TEI = total energy intake

[a]Low and high categories are divided at the weighted median among the exposed group within the BPA subpopulation (n = 8,789)

[b]Adjusted for age, sex, race/ethnicity, BMI, PIR, NHANES survey cycle, and urinary creatinine

[c]Model 1 with additional adjustment of intake (% of TEI) from non-fast food group counterpart (e.g. fast food dairy intake adjusted for non-fast food dairy intake, fast food egg intake adjusted for non-fast food egg intake, etc.)

[d]Model 1 with additional adjustment for fast food intake (% of TEI) of all other food groups

Table S3. Association between recent fast food consumption and urinary concentrations of DiNPm stratified by age group in the US general population, NHANES 2005-2010 (n = 6,629) [a,b]

	Children (6-11 years) (n = 956)		Adolescents (12-19 years) (n = 1,298)		Adults (≥20 years) (n = 4,375)	
	n	Percent difference (95% CI)	*n*	Percent difference (95% CI)	*n*	Percent difference (95% CI)
Fast food intake (% of TEI)[c]						
None (0)	621	Referent	754	Referent	2979	Referent
Low (0.08 - 34.8)	191	13.5 (-3.3, 33.2)	235	37.7 (8.4, 74.9)**	688	26.8 (11.5, 44.1)**
High (34.9 - 100)	144	-5.0 (-23.7, 18.3)	309	39.4 (13.2, 71.6)**	708	50.0 (29.4,73.8)**
p for trend		0.84		0.0008		<0.0001

**p<0.01

Abbreviations: DiNPm = DiNP metabolite (MCOP); TEI = total energy intake

[a]Adjusted for sex, race/ethnicity, BMI, PIR, NHANES survey cycle, and urinary creatinine

[b] p = 0.021 for fast food intake*age group multiplicative interaction term

[c]Low and high categories are divided at the weighted median of the exposed population (n = 8,877)

Table S4. Association between recent fast food consumption and urinary concentrations of ∑DEHPm stratified by race/ethnicity in the US general population, NHANES 2003-2010 (n = 8,877) [a,b]

	Hispanic (including Mexican Americans) (n = 2,681)		NH White (n = 3,997)		NH Black (n = 2,199)	
	n	Percent difference (95% CI)	*n*	Percent difference (95% CI)	*n*	Percent difference (95% CI)
Fast food intake (% of TEI)[c]						
None (0)	1760	Referent	2748	Referent	1274	Referent
Low (0.08 - 34.8)	455	0.2 (-12.5, 14.7)	652	18.1 (6.4, 31.0)**	393	20.9 (6.8, 37.0)**
High (34.9 - 100)	466	16.0 (-1.3, 36.3)	597	20.2 (5.7, 36.7)**	532	49.8 (31.5,70.7)**
p for trend		0.09		0.001		<0.0001

**p<0.01

Abbreviations: ∑DEHPm = molar sum of four DEHP metabolites; NH = Non-Hispanic; TEI = total energy intake

[a]Adjusted for sex, age, BMI, PIR, NHANES survey cycle, and urinary creatinine

[b] p = 0.037 for fast food intake*race/ethnicity interaction term

[c]Low and high categories are divided at the weighted median of the exposed population (n = 8,877)
